# Air Pollution Exposure and Covid-19 in Dutch Municipalities

**DOI:** 10.1007/s10640-020-00491-4

**Published:** 2020-08-04

**Authors:** Matthew A. Cole, Ceren Ozgen, Eric Strobl

**Affiliations:** 1 Department of Economics, University of Birmingham, Birmingham, B15 2TT UK; 2 IZA, Bonn, Germany; 3 University of Bern, Bern, Switzerland

**Keywords:** Covid-19, Air pollution, Netherlands, Spatial spillovers, I21, I23, Q53

## Abstract

In light of the existing preliminary evidence of a link between Covid-19 and poor air quality, which is largely based upon correlations, we estimate the relationship between long term air pollution exposure and Covid-19 in 355 municipalities in the Netherlands. Using detailed data we find compelling evidence of a positive relationship between air pollution, and particularly $PM_{2.5}$ concentrations, and Covid-19 cases, hospital admissions and deaths. This relationship persists even after controlling for a wide range of explanatory variables. Our results indicate that, other things being equal, a municipality with 1 μg/m^3^ more $PM_{2.5}$ concentrations will have 9.4 more Covid-19 cases, 3.0 more hospital admissions, and 2.3 more deaths. This relationship between Covid-19 and air pollution withstands a number of sensitivity and robustness exercises including instrumenting pollution to mitigate potential endogeneity in the measurement of pollution and modelling spatial spillovers using spatial econometric techniques.

## Introduction

The Covid-19 pandemic is causing significant social and economic impacts across large parts of the world. At the time of writing the number of Covid-19 cases worldwide has reached 7.2 million, while the death toll has exceeded 400,000.^1^ Governments and health care systems are facing the immense challenge of trying to control the spread of the virus and to prevent hospitals from being overwhelmed as millions of individuals remain subject to lockdown and face significant economic uncertainty. In order to respond to these unprecedented challenges it is important for policy makers and health care professionals to understand which groups of individuals suffer the highest morbidity and mortality risks from Covid-19 and which factors may exacerbate these risks.

A contributory factor that has been tentatively explored in several recent academic studies is poor air quality. While some such studies have identified the significant improvements in air quality that have resulted from Covid-19 lockdowns (Cicala et al. 2020; Cole et al. 2020), others have pointed to a correlation between Covid-19 hotspots and areas with high levels of pollution concentrations (Travaglio et al. 2020; Conticini et al. 2020). It is well known that long term exposure to pollutants such as nitrogen dioxide ($NO_{2}$), sulphur dioxide ($SO_{2}$), and fine particulate matter ($PM_{2.5}$) contributes to cardiovascular disease, reduces lung function, and causes respiratory illness (Faustini et al. 2014; Ming Han et al. 2015; Katanoda et al. 2011; Abbey et al. 1999; De Weerdt et al. 2020). These pollutants have been shown to cause a persistent inflammatory response even in the relatively young, and to increase the risk of infection by viruses that target the respiratory tract (Travaglio et al. 2020; Conticini et al. 2020). While Covid-19 produces only mild symptoms for most sufferers, in a minority of cases it results in an excessive inflammatory response causing Acute Respiratory Distress Syndrome (ARDS) and death. Given the clear overlaps between the symptoms of Covid-19-induced ARDS and long term exposure to air pollution, a number of studies have begun to explore the links between the two.

Focusing on the UK, Travaglio et al. (2020), for instance, find evidence of a correlation between Covid-19 cases and concentrations of nitrogen oxides and ozone, while Ogen (2020) examines 66 regions across Italy, France, Germany, and Spain and finds similar evidence. Conticini et al. (2020) focus on Northern Italy and conclude that pollution concentrations are a likely contributor to the high Covid-19 death rates experienced in that region. Setti et al. (2020) find similar evidence for Italy and raise the possibility that particulate matter could actually carry the virus thereby directly contributing to its spread. A preliminary analysis of the Netherlands also finds evidence of a link between concentrations of $PM_{2.5}$ and Covid-19 cases (Andree 2020). Finally, Wu et al. (2020) examine US counties and estimate the relationship between county-level Covid-19 death rates and long-term concentrations of fine particulate matter ($PM_{2.5}$) using a negative binomial count model, controlling for a wide range of confounding factors. They conclude that a 1 μg/m^3^ increase in $PM_{2.5}$ is associated with an 8% increase in the Covid-19 death rate.

While the above studies provide useful preliminary evidence, Conticini et al. (2020) and Ogen (2020) offer only geographical correlations between Covid-19 cases and pollution exposure, whereas Travaglio et al. (2020) take a similar approach but control only for differences in population density and do so across only 7 relatively large regions. Establishing a convincing link between exposure to pollution and Covid-19 cases requires individual-level data with the ability to control for individual characteristics, such as age and the presence of underlying health conditions.^2^ Since individual-level data on Covid-19 infections is not available, the next best alternative is to examine a large number of small geographic regions with detailed data on the characteristics of those regions. This allows the researcher to assess whether any correlation between Covid-19 and pollution exposure still holds once differences in social deprivation, population density, ethnic composition, and other factors are controlled for. While Wu et al. (2020) come closest to doing this, US counties are still relatively large, raising the question of how well such aggregated data can capture the local variation in confounding effects without being ‘averaged out’. Furthermore, by focusing only on particulate matter, and on Covid-19 deaths, it is not clear whether other pollutants have an effect on Covid-19 deaths once other factors are controlled for or, indeed, on Covid-19 infections or hospitalisation rates.

With the above in mind, this paper undertakes a detailed analysis of the relationship between pollution concentrations and Covid-19 using data for 355 relatively small Dutch municipalities. More specifically, by using high-resolution air pollution data as well as combining administrative and municipal-level data, we estimate the relationship between long term exposure to concentrations of $PM_{2.5}$, $NO_{2}$, and $SO_{2}$ and the number of Covid-19 infections, the number of individuals hospitalised with Covid-19, and the number of those who died as a result of Covid-19. We use a negative binomial count model and are able to control for a wide range of potential confounding effects, including those relating to income, age, underlying health conditions, education, social deprivation, ethnic composition, workplace characteristics, spatial and social proximity to potential risk factors and others. Our analysis utilises Covid-19 data up to 5th June 2020 allowing us to capture almost the full wave of the epidemic and hence much more fully than the previous studies which have examined data up to only March or early April. Finally, we undertake a number of robustness exercises including instrumenting pollution to address some possible endogeneity concerns. Therefore, compared to Wu et al. (2020) we use data at a finer resolution^3^ and include more controls, plus mitigate concerns arising from imprecise measurement of pollution.

The Netherlands provides a useful setting in which to examine the link between Covid-19 and air pollution. As a relatively small, densely populated nation with an ethnically diverse, aging population, the country faces a number of potential Covid-19 risk factors. It also shares an open land border with Germany and Belgium, the latter a country that currently has the highest number of Covid-19 deaths per capita. The Netherlands additionally experiences hot spots of poor local air pollution both within urban areas and also, in the case of $PM_{2.5}$, in more rural areas perhaps due in part to intensive livestock farming. By early June 2020, the Netherlands had experienced over 6000 deaths as a result of Covid-19, resulting in the 7th highest number of Covid-19 deaths per capita.^4^

Our results provide compelling evidence of a statistically significant positive relationship between air pollution and Covid-19 cases, hospital admissions and deaths. More specifically we find that, on average, and other things being equal, a municipality with 1 μg/m^3^ more $PM_{2.5}$ concentrations than another will have between 9.4 and 15.1 more Covid-19 cases, depending on our model. It will also have between 2.9 and 4.4 more Covid-19 hospital admissions and between 2.2 and 2.8 more Covid-19 deaths.

The remainder of this paper is organized as follows. Section 2 provides background information on Covid-19 and air pollution in the Netherlands and Sect. 3 presents our empirical methodology. Section 4 describes our data, Sect. 5 reports our results and Sect. 6 concludes.

## Covid-19 and Air Pollution

The first confirmed Covid-19 case in the Netherlands occurred in late February 2020, and by early June over 46,000 cases had been identified. Daily new cases of Covid-19 peaked at 1,335 on April 10th, while the daily death toll peaked 3 days earlier at 235. Both daily new cases and daily deaths have been declining steadily since. While all 12 of the Netherlands’ provinces have experienced broadly similar trends, levels of Covid-19 cases have differed significantly across provinces. For instance, in the southern provinces of North Brabant and South Holland daily new cases peaked at over 350,^5^ while in other provinces such as Groningen and Drenthe, both in the north of the country, new cases peaked at fewer than 40 per day.^6^

**Fig. 1:**
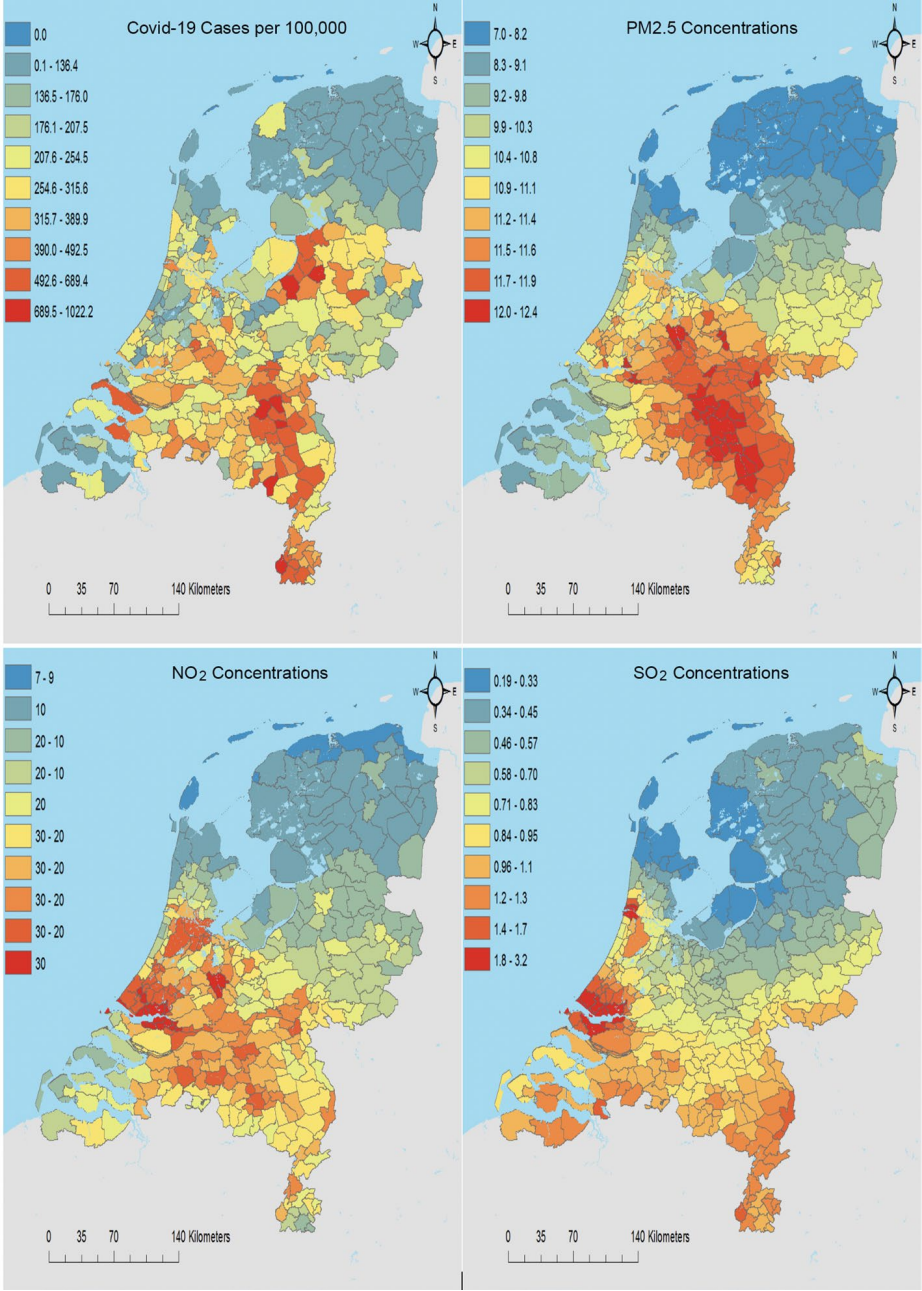
Covid-19 cases per 100,000 people and annual concentrations of $PM_{2.5}$, $NO_{2}$ and $SO_{2}$ averaged over the period 2015–19

From the outset of the epidemic within the Netherlands the south east has experienced a disproportionate number of Covid-19 cases. Figure 1 shows the distribution of cases per capita across the 355 municipalities of the Netherlands up to June 5th 2020. The red ‘hotspots’ in the south east, which are largely within the provinces of North Brabant and Limburg, demonstrate the relative intensity of cases in those regions. Unusually, these are relatively rural regions with low population density raising the question of why these provinces have been so badly affected by Covid-19.^7^

One potential explanation raised by the Dutch media has been the annual carnival celebrations held during the last week of February and beginning of March which were concentrated largely within North Brabant and Limburg. These celebrations attracted thousands of people from all over the country to street parties and parades, as they do each year. Numerous Dutch media reports have suggested that these celebrations may at least partially explain the rapid spread of Covid-19 within these regions and also to other regions of the Netherlands via carnival participants.^8^ However, the significant variation in Covid-19 cases across the municipalities within these two provinces suggests that the carnival is unlikely to fully explain the density and distribution of these cases.

**Fig. 3:**
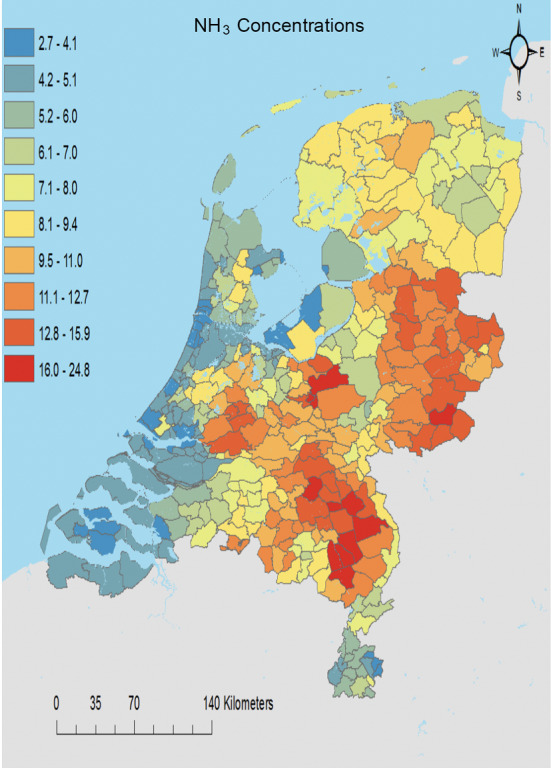
Ammonia ($$NH_{3}$$) concentrations (2019)

Another possible explanation that has received less attention in the Netherlands revolves around the intensive livestock farming that takes place within North Brabant and Limburg. These regions house over 63% of the Netherlands’ 12 million pigs and 42% of its 101 million chickens.^9^ Such intensive livestock production produces large quantities of ammonia ($NH_{3}$), which can be an important contributor to $PM_{2.5}$ concentrations. Figure 3 in the “Appendix” provides a map of 2019 $NH_{3}$ concentrations and shows that the south eastern regions have some of the highest concentrations within the Netherlands. Indeed, a comparison with the map of $PM_{2.5}$ concentrations in Fig. 1 shows a very similar pattern illustrating how ammonia potentially contributes to $PM_{2.5}$.

While the maximum annual average concentration of $PM_{2.5}$ at municipality level is 12.3 μg/m^3^, the equivalent figure for a 1 km × 1 km gridcell is 23.9 μg/m^3^, which is close to the EU air quality standard of 25 μg/m^3^. This indicates that local concentrations of $PM_{2.5}$ within the regions of North Brabant and Limburg are close to dangerous levels even when averaged annually, raising the likelihood that for shorter periods they may exceed safe levels. Since $PM_{2.5}$ concentrations show a similar spatial distribution to Covid-19 cases, also in Fig. 1, it’s reasonable to examine this potential link and to see whether the visual relationship between Covid-19 cases and $PM_{2.5}$ concentrations withstands controlling for other potential contributory factors.^10^

## Methodology

In order to examine the relationship between Covid-19 and air pollution we begin by estimating Eq. 1 for 355 municipalities:

1 $$\begin{aligned} \begin{aligned} C_{i} = \phi pollution_{i} + \beta _1 D_{i}^{'} + \beta _2 P_{i}^{'} + \beta _3 E_{i}^{'}+ \beta _4 S_{i}^{'} + \beta _5 H_{i}^{'} + \gamma _{r} + \epsilon _{i} \end{aligned} \end{aligned}$$

where *C* refers to Covid-19 cases, the number of individuals hospitalised by Covid-19, or the number of deaths from Covid-19 in municipality *i* as of June $5^{th}$ 2020. *Pollution* refers to annual concentrations of $PM_{2.5}$, $NO_{2}$, or $SO_{2}$, averaged over the period 2015-2019. Vectors $D^{'}$, $P^{'}$, $E^{'}$, $S^{'}$ and $H^{'}$ contain control variables capturing demography, social and physical proximity, employment/education, spatial and health variables, respectively, for the year 2019, as defined in the next section. The term $\gamma _{r}$ denotes province level fixed effects for each of our 12 provinces,* r*.

Since our dependent variables take the form of discrete count variables, estimating Eq. 1 using OLS could result in inconsistent, inefficient, and biased estimates (Long 1997; Hoffman 2004). The alternative is to use Poisson or negative binomial count models. Since the error assumption of the Poisson model requires the conditional mean to equal the conditional variance, a condition that our over-dispersed data fails to meet, we employ the negative binomial model. This model builds upon the Poisson model by adding a parameter that allows the conditional variance to exceed the conditional mean.

Importantly our attempt to estimate the relationship between pollution exposure and Covid-19 cases (as well as hospital admissions and deaths) potentially suffers from a number of econometric challenges:

1. *Omitted variable bias*
  If we fail to control for all potential determinants of Covid-19 that are possibly correlated with pollution, then the estimated coefficients on the pollution measures could potentially be biased. As will be outlined in Sect. 4, our strategy is to limit omitted variable bias by including a wide range of control variables. However, clearly omitted variable bias still persists and therefore we make no claim to be addressing causal relationships.
  In principle our pollution variables may be subject to systematic measurement error if the error was in some way related to the pollution-Covid-19 relationship. For instance, if individuals with poor health were more (or less) likely to live in polluted areas and hence were more (or less) likely to catch or succumb to the virus. We are unable to identify any potential mechanisms of this nature and our wide range of explanatory variables should obviously help to control for any such effects if they were to exist.
2. *Non-systematic measurement error of pollution*
  Such measurement error could arise if we are not accurately capturing long-term pollution exposure within each municipality. While Sect. 4 outlines how we measure long-term pollution exposure, we note that any non-systematic measurement error of this kind is likely to result in a conservative estimate of the association between pollution and Covid-19 due to possible attenuation bias.
  Nevertheless, we do instrument pollution in Eq. 1 as a means of further reducing concerns around attenuation bias. Since we are using a non-linear count model we instrument using a control function approach, which is likely to be more efficient than a standard instrumental variables model (Wooldridge 2015). This approach involves estimating air pollution, our potentially endogenous variable, as a function of our instruments and other exogenous variables, and then inserting the predicted errors from this first stage into the second stage as a separate control variable (in addition to our air pollution variable).^11^ A simple test of the statistical significance of the coefficient on the predicted residuals will inform us whether our pollution variable was indeed endogenous, a procedure equivalent to a Durbin–Wu–Hausman exogeneity test.
  To be suitable for use as an instrument a variable should be correlated with air pollution but independent of our Covid-19 variables and hence should only influence them through the effect of air pollution. We experiment with two potential candidates. The first is a long lag of air pollution. More specifically, we use annual pollution concentrations averaged over the period 1995–2000. Second, we use a measure of the average commuting time for residents in each municipality. This variable draws upon free-flow road network data from VUGeoPlaza enabling us to calculate average travel time for every *j* and *k* location pair. In order to calculate the commuting times we obtain the residential and work locations of each worker at the $4-digit$ postal code level (which corresponds to neighbourhood level) by constructing an employer-employee data set (LEED) based on linking administrative data, Dutch Labour Force surveys, and Tax Registers.^12^ We then combine the travel time data with the LEED. Travel times linked with the LEED allow us to calculate the commuting time of each worker to their actual place of work. Additionally we calculate the travel time based on the Euclidean distance between the $4-digit$ postal code, assuming an average speed of 10 km/h. We then choose the lowest of the commuting time and the Euclidean distance travel time for each worker and average these across municipalities.^13^ Differences in commuting time between municipalities reflect differences in the duration and intensity of journeys undertaken and should therefore influence differences in air pollution concentrations. We see no reason why commuting time should directly influence Covid-19 cases.
3. *Measurement error of Covid-19*
  A third potential econometric challenge is the existence of possible measurement error within our Covid-19 data. Non-systematic measurement error would tend to reduce the precision of our estimates by increasing the standard errors on the estimated coefficients. Alternatively, if the measurement error is related to other omitted variables that are important for the pollution-Covid-19 relationship, or to its own values, this could be a cause for concern. Section 4 discusses the nature of our Covid-19 data and its potential deficiencies in more detail.
4. *Spillover effects*
  The final potential econometric challenge is the possible existence of spillover effects caused by the virus spreading from one municipality to another. To allow for this possibility we extend Eq. 1 to include a spatially lagged dependent variable and a spatial error term, each using spatial weight matrices with varying distance cut-offs, as specified in Eq. 2.
  2 $$\begin{aligned} \begin{aligned} C_{i} = \theta pollution_{i} + \alpha _1 D_{i}^{'} + \alpha _2 P_{i}^{'} + \alpha _3 E_{i}^{'}+ \alpha _4 S_{i}^{'} + \alpha _5 H_{i}^{'} + \rho w_{i}C_{i} + \lambda w_{i}\epsilon _{i} + \mu _{i} \end{aligned} \end{aligned}$$
  where ${w_{i}}$ is an inverse distance spatial weight matrix with a 50 km cut-off, a 100 km cut-off, or no cut-off at all. All other variables are as previously specified. Note that the inclusion of a spatially lagged dependent variable into a non-linear count data model is not straightforward and hence there has yet to be a widely accepted method of doing so (Glaser 2017). Equation 2 is therefore a linear model estimated using maximum likelihood.

## Data and Summary Statistics

### Data

We utilize extensive municipality-level data combined with administrative micro-data and spatial data from various sources in the Netherlands. Our analysis rests on municipality level cross-sectional Covid-19 data provided by the National Institute for Public Health and the Environment (RIVM) and a rich set of controls from 2019 (unless otherwise stated). We further use high resolution spatial data for air pollution indicators which span the period 2015–19.

The municipal-level data is obtained from the data repository *Statline* of the Statistics Netherlands, while some variables are aggregated from the administrative micro-data up to municipality level. When modelling the relationship between Covid-19 and air pollution, we control for a wide range of potentially confounding effects which we categorise as demographic, social and physical proximity, employment and education, spatial, and health-related. Below we discuss Covid-19 and pollution data and each category of control variable:^14^

#### Covid-19

The Covid-19 data used in our analysis is obtained from RIVM. The Regional Public Health Service Centres (known as GGD) from all across the country^15^ provide RIVM with the Covid-19 data, which is then corrected for any inconsistencies before being released by RIVM. As stated by RIVM on their website, there is a slight delay between the day of hospitalization or death and the day on which the the number of cases is reported.^16^ However, when the data is available RIVM does the necessary adjustment to the data retrospectively. This incident of slight delay is well-known for all countries. Again similar to the other countries, the number of Covid-19 cases is likely to be an underestimate of the true number of cases as not everybody in the country is tested. In addition, the disparity between the official Covid-19 death toll and calculations of excess deaths suggests that official statistics are under-reporting the true death toll, as seems to be happening world-wide.^17^ Finally, it is important to note that all Covid-19 variables are coded by the residence of each individual rather than the address of the test centre or the hospital to which they are admitted.

#### Air Pollution

Annual concentrations of $PM_{2.5}$, $NO_{2}$, and $SO_{2}$ measures are provided by RIVM. These pollution concentrations are reported at the level of $1\times 1\,{\text {km}^2}$ grid-cells having been modelled using a wide range of sources and components in the Netherlands and in other European countries. Maps of the spatial distribution of pollution concentrations were calculated using the Operational Priority Substances dispersion model, which constructs the average annual concentrations of pollutants stemming from the dispersion, transport, chemical conversion and dispersion of emissions. The resultant concentrations are calibrated with the observations from the Dutch Air Quality Monitoring Network (Velders et al. 2020; Fischer et al. 2020).^18^

To produce municipality level measures of pollution concentrations we use the median grid-cell concentrations within each municipality. In order to overcome any potential measurement bias due to annual fluctuations and to capture the long term exposure of residents within a municipality, we average the annual pollution concentrations data over the 5-year period 2015–2019 (in our sensitivity analysis we also use a 10 year average 2010–19).^19^

#### Demographic and Labour Market Indicators

The demographic controls include the total population as well as the density of population in each municipality, expressed as population per square kilometre. The share of the population over 70 years of age in our regressions accounts for the particular vulnerability of the elderly to Covid-19, as the stylized facts from around the world indicate higher casualties among the elderly population. We also include the share of the population under 18 years old, with the omitted category being the working age share of the population.

Finally, several recent studies from a number of countries including the UK and the US^20^ have indicated that ethnic minorities are disproportionately affected by Covid-19. There are a number of potential reasons why this might be so. First, Zorlu and Hartog (2012) show that ethnic groups in the Netherlands are more likely to work in manual occupations, which have been shown to increase the risk of exposure to Covid-19 due to the greater frequency of face-to-face contact amongst workers (Lewandowski 2020).^21^ Second, ethnic minorities may be more likely to experience social deprivation, live in smaller housing and belong to larger households. As will be discussed below, we try to control for each of these factors by taking housing and household size conditions into account. Other risk factors may include the use of certain cultural practices (e.g., attending places of worship) or having a disposition for underlying health conditions. To control for any such effects we include the number of non-western migrants as a share of the population. Non-western migrants in the Netherlands are defined as the number of immigrants who are born in Africa, Latin America, Asia (excluding Japan and Indonesia), and Turkey.

We also include the average household income level and the share of employment in elementary occupations to control for the possibility that groups that are economically and occupationally more vulnerable could be more affected by Covid-19. Workers in elementary occupations are perhaps more likely to work in close proximity to fellow workers and were perhaps less likely to be able to work from home once the epidemic began. Finally, our specification includes the number of highly educated people as a share of the total educated population at the municipal level. Highly educated is defined as those who have obtained a bachelor’s degree or higher.

#### Social and Physical Proximity

While the majority of countries have disproportionately experienced Covid-19 infections in large metropolitan areas, the empirical findings so far are unclear about the role played by population density.^22^ Epidemiological studies have argued that density *per se* is not important but rather it is the type of *interaction* between people that is important e.g. weddings, close family events and occasions when individuals come into close proximity with each other (Hu et al. 2013). One such occasion in the Netherlands is the annual Carnival which is widely celebrated in the southern regions of the country—North Brabant and Limburg—as discussed in Sect. 2. To capture the potential impact of the Carnival we initially rely on province level fixed effects which capture the effects in North Brabant and Limburg and all other provinces individually. Furthermore, in sensitivity analysis we test a Carnival(municipality) measure by creating a dummy variable for the seven most populous municipalities (Den Bosch, Tilburg, Breda, Bergen op Zoom, Eindhoven, Helmond, Oss) within the borders of North Brabant and Limburg provinces. Alongside this, in order to account for the potential spread of the virus from the areas where the Carnival is widely celebrated, we also test a proximity to Carnival(municipality) measure that is the inverse distance from the centroid of these seven Carnival municipalities to the centroid of all municipalities in the Netherlands.

To capture physical interactions between households we include the share of small housing, defined as the share of total housing that is between 15 and 50 ${m^2}$ in size,^23^ and the average household size. We also apply the same principle to workplaces. We aggregate a linked employer-employee data set based on the population of firms and employees in the Netherlands from 2016 records in order to create an average firm size variable at the municipal level. This data is obtained from Tax Registers, which include approximately 12 million observations from the universe of employees.

#### Spatial Controls

Our specification includes several spatial variables which capture the spatial interactions and spillovers between municipalities. First, we account for the municipalities where Covid-19 infections may have been influenced by the transmission of the virus beyond Dutch national borders. The Dutch municipalities bordering Belgium experience substantial daily cross-border mobility and Covid-19 cases in Belgium have been the highest of all countries in per capita terms. Dutch municipalities that border Germany also experience intensive daily crossings, although, in contrast, the Covid-19 cases in Germany had been relatively low compared to other European countries. In order to capture the potential contagion through cross-border mobility, we therefore construct 2 dummy variables; municipalities bordering Germany and municipalities bordering Belgium.

Second, Schiphol airport within the borders of the municipality of Haarlemmermeer (9 km from Amsterdam) is the main international hub of the country and one of the largest airports in Europe with approximately 72 million passengers a year. We therefore construct a variable capturing the inverse distance from each municipality centroid to Schiphol airport to capture the potential contagion from the high volume of passengers. Lastly, to capture the effect of the prevailing wind from the North Sea which could mean less physical spread of the virus and/or reduced spillovers from neighbours, we include a dummy variable for all of the municipalities on the Dutch coast.

#### Health-Related

Covid-19 statistics in many countries have indicated that those persons with underlying health conditions are likely to be disproportionately affected by the virus. Our data allows us to control for these groups at the municipality level. We include in our estimations the share of smokers and the share of those suffering from obesity in the total local population in 2019. Moreover, the share of people receiving incapacity benefits is also included to proxy those who cannot be active in the labour market due to health constraints. In unreported estimations, to capture the supply of medical services we tested the number of hospitals within 5 km, 10 km and 20 km radii of each municipality centroid. In each case these variables were not statistically significant. They were omitted as they were highly correlated with population density (the correlation was between 0.63 and 0.67).

### Descriptive Statistics

We report the descriptive statistics of our data in Table 1. Our Covid-19 data covers the period since the first Covid-19 case in February until the tail-end of the epidemic’s wave in June. On average, each municipality has experienced a total of 131 Covid-19 cases over this period (equivalent to 2690 per million of population) with a maximum number of 2416 (equivalent to 49,628 per million). Over the same period on average 33 hospital admissions were recorded across all municipalities (maximum 611) while on average 17 people died (maximum 336).^24^

Our data set includes 355 municipalities based on the 2019 municipal borders in the Netherlands, where each has an average population of approximately 47,000 people, of which 7.4% are non-Western European immigrants. Although the size of the municipalities is broadly uniform, average population density across municipalities shows a large variation, ranging from 23 individuals per km^2^ to 6523 per km^2^. On average 3.5% of housing is classed as small (15–50 m^2^) while the average household size is 2.3 individuals. Finally, 15% of the population is over 70 years old.

The median annual wage is 32,722. With 32% of the population holding a university degree or higher, Netherlands has a relatively highly-educated population. 9% of the population performs elementary occupations which require low-skilled manual-task intensive work. Median firm size is 556 workers, but becomes larger for peripheral areas, although the average firm size in large cities is still around a standard deviation higher than the median. Although known to be one of the healthiest nations, 19% of the population are smokers and 14% experience obesity (defined as % of over 19 s with BMI>30 kg/m$^{2}$). 6% receive incapacity benefits due to being unable to work.

With regard to our pollution concentrations data which is measured in μg/m^3^, we see that the average value of $PM_{2.5}$ concentrations in our dataset is 10.49, with a maximum of 12.26. For $NO_{2}$ concentrations the mean value is 15.76, with a maximum value of 27.41, and for $SO_{2}$ concentrations the mean value is 0.80 with the maximum being 3.09. While the EU air quality standards do not stipulate a safe limit for annual concentrations of $SO_{2}$, for $PM_{2.5}$ and $NO_{2}$ concentrations the limit is 25 μg/m^3^ and 40 μg/m^3^, respectively. We can therefore see that 5 year averages of annual concentrations in the Netherlands do not exceed these limits. However, it is worth noting that the maximum values of the annual concentrations of our 1 × 1 km^2^ grid-cells that form the basis of our municipality pollution data are 23.9 μg/m^3^ and 62.4 μg/m^3^ for $PM_{2.5}$ and $NO_{2}$, respectively. In the case of $NO_{2}$, this is substantially beyond the EU safe limit, while for $PM_{2.5}$ it is very close to the safe limit.

**Table 1:** Summary statistics

Variable	Mean	SD	Minimum	Maximum
PM2.5 5 year ave	10.49	1.35	6.92	12.26
NO2 5 year ave	15.76	3.86	6.84	27.41
SO2 5 year ave	0.80	0.36	0.21	3.09
Covid-19 cases	131.46	224.89	0	2416
Covid-19 hospital admissions	33.02	55.13	0	611
Covid-19 deaths	16.87	31.45	0	336
Carnival	0.02	0.14	0	1
Proximity to carnival	0.00	0.00	0	0.0001
Proximity to Schiphol	86.49	46.15	5.27	194.83
Belgian border	0.073	0.26	0	1
Coast	0.093	0.29	0	1
German border	0.09	0.29	0	1
Ave gross income	32.72	3.05	23.50	53.6
ln (firm size)	8.9	0.51	3.26	10.06
Sh elementary occup	0.089	0.031	0	0.20
Sh high educated	0.32	0.095	0.071	0.90
Ave household size	2.30	0.18	1.71	3.34
Sh of small houses	0.036	0.045	0	0.67
Sh pop under 18	0.20	0.024	0.13	0.34
Sh pop over 70	0.15	0.025	0.063	0.24
Obesity	14.39	2.13	9	22
Smokers	19.62	2.75	14	31
Sh on incapacity benefits	0.063	0.017	0.02	0.13
Pop density	877.32	1042.72	23.11	6523.14
Ln (population)	10.40	0.83	6.84	13.67
Sh non-western migrants	0.074	0.059	0.014	0.39
Ave commuting time	14.07	8.29	4.28	134.43

The proximity to carnival measure is calculated as the negative exponential of distance i.e. (exp(-distance)) and produces numbers that are very small in magnitude, thereby explaining the large estimated coefficients for this variable in our regression results

## Results

Table 2 provides our initial negative binomial estimates of Eq. 1 with columns (1)–(3) providing the estimates of Covid-19 cases for the three pollutants. Columns (4)–(6) do the same for hospital admissions, and columns (7)–(9) provide estimates of Covid-19 deaths, again for the three pollutants.

Focusing initially on the estimated pollution coefficients, we see that $PM_{2.5}$ concentrations have a positive and statistically significant relationship with Covid-19 cases, hospital admissions and deaths. In the case of $NO_{2}$ we find a positive and statistically significant association between Covid-19 cases and deaths, but this is not statistically significant for hospital admissions. Finally, for $SO_{2}$ we again discover a positive relationship with our dependent variables, but this is not statistically significant. Using a Fisher combination test across the specifications for the different dependent variables for each pollutant suggests that both $PM_{2.5}$ and $NO_{2}$ have a significant impact on Covid-19 outcomes in general, but $SO_{2}$ does not.^25^

After calculating marginal effects, we find that a one unit increase in a municipality’s $PM_{2.5}$ concentrations is associated with 9.4 more Covid-19 cases, 3.0 more hospital admissions, and 2.3 more deaths within that municipality. A one unit increase in $NO_{2}$ increases a municipality’s Covid-19 cases by 2.2 and deaths by 0.35. To make these comparable, a one standard deviation increase in $PM_{2.5}$ and $NO_{2}$ concentrations increases Covid-19 cases by 12.7 and 8.5, respectively. The same one standard deviation in $PM_{2.5}$ and $NO_{2}$ concentrations increases Covid-19 deaths by 3.1 and 1.4, respectively. Table 3 summarises our pollution marginal effects from the estimations in which pollution is statistically significant.

Turning to the other explanatory variables, and focusing on those that are generally statistically significant, we find that municipalities next to the German border have lower Covid-19 cases, hospital admissions, and deaths compared to other municipalities. We also find average household income to have a negative association with all three dependent variables, while average household size and the share of housing that is small both have positive relationships with our Covid-19 variables. Relative to the share of the working age population, the share of those under 18 has a negative association with Covid-19 while, as expected, the share over 70s has a positive association. Finally, smokers, the share of non-Western immigrants, and the total population of each municipality are associated with increased Covid-19 cases, hospital admissions, and deaths.

Since we find the greatest statistical significance and largest marginal effects for $PM_{2.5}$ concentrations, for reasons of space we focus on this pollutant alone for the remainder of our analysis, although the results for other pollutants can be found in the “Appendix”. Table 4 reports the results of the instrumental variable estimations using a control function approach, as outlined previously. In Table 4 we see that the estimated coefficients on $PM_{2.5}$ concentrations remain positive and statistically significant, with a similar magnitude to those in Table 2. It is notable that the first stage residuals are not statistically significant, thus failing to reject the null of exogeneity. One can also see that an F-test on our instruments in the first stage is highly statistically significant. Table 8 in the “Appendix” reports the first stage results, which use commuting time, commuting time squared, and lagged particulate matter concentrations from 1995 to 2000 as instruments. In sensitivity analysis we tested different combinations of these variables including lagged pollution alone, commuting time alone, and commuting time in linear form only. In each case the first stage residuals were not statistically significant in the second stage. Our results therefore provide reassurance that our estimations in Table 2 are not being unduly influenced by endogeneity arising from attenuation bias.

**Table 2:** Main estimation results

	Covid-19 cases			Hospital admissions			Deaths		
	(1)	(2)	(3)	(4)	(5)	(6)	(7)	(8)	(9)
PM2.5, 5 year ave	0.11* (0.051)			0.15* (0.065)			0.23** (0.073)		
NO2, 5 year ave		0.027* (0.012)			0.015 (0.013)			0.035* (0.016)	
SO2, 5 year ave			0.11 (0.079)			0.055 (0.065)			0.18 (0.10)
Belgian border	0.18 (0.11)	0.16 (0.11)	0.14 (0.11)	− 0.079 (0.11)	− 0.11 (0.12)	−0.12 (0.12)	0.084 (0.16)	0.041 (0.16)	0.018 (0.16)
German border	− 0.29*** (0.084)	− 0.29*** (0.080)	− 0.28*** (0.081)	− 0.25** (0.081)	− 0.23** (0.080)	− 0.23** (0.082)	− 0.36*** (0.11)	− 0.34*** (0.10)	− 0.34*** (0.11)
Ave gross income	− 0.027* (0.012)	− 0.022* (0.011)	− 0.020* (0.011)	− 0.027* (0.013)	− 0.019 (0.012)	− 0.018 (0.012)	− 0.047* (0.022)	− 0.035 (0.021)	− 0.032 (0.021)
Ave household size	1.80*** (0.38)	1.73*** (0.37)	1.69*** (0.37)	2.19*** (0.50)	2.05*** (0.50)	2.02*** (0.50)	2.00** (0.68)	1.82** (0.68)	1.76** (0.67)
Sh of small houses	1.24*** (0.27)	1.27*** (0.26)	1.28*** (0.27)	0.68* (0.28)	0.70* (0.28)	0.71* (0.28)	0.87* (0.37)	0.94** (0.35)	0.97** (0.35)
Sh of pop under 18	− 3.26 (2.29)	− 3.10 (2.29)	− 3.06 (2.28)	− 8.02** (2.82)	− 7.76** (2.86)	− 7.73** (2.86)	− 2.76 (3.74)	− 2.18 (3.76)	− 2.00 (3.72)
Sh of pop 70+	4.60** (1.54)	4.22** (1.45)	3.59** (1.50)	1.95 (1.63)	1.03 (1.59)	0.64 (1.66)	9.15*** (2.32)	8.00*** (2.21)	7.16** (2.24)
Smokers	0.057*** (0.017)	0.052** (0.017)	0.049** (0.017)	0.033* (0.015)	0.025 (0.016)	0.024 (0.016)	0.063* (0.021)	0.050* (0.024)	0.046 (0.024)
Ln (population)	0.99*** (0.041)	0.99*** (0.040)	0.99*** (0.042)	1.00*** (0.052)	0.99*** (0.051)	0.99*** (0.052)	1.02*** (0.066)	1.00*** (0.065)	1.01*** (0.068)
Sh non-west migrants	1.40* (0.65)	1.34* (0.64)	1.37* (0.66)	1.84** (0.62)	1.91** (0.62)	1.93** (0.61)	2.24* (1.04)	2.32* (1.01)	2.23* (0.98)
Observations	355	355	355	355	355	355	355	355	355
Province dummies	YES	YES	YES	YES	YES	YES	YES	YES	YES
Pseudo R^2^	0.17	0.17	0.17	0.19	0.19	0.19	0.18	0.18	0.18

Robust SE in parentheses ***p < 0.001; **p < 0.01; *p < 0.05. Additional controls included in the estimations but not reported are proximity to Schiphol airport, coastal dummy, average firm size, share of workers in elementary occupations, share of obesity, population density, share of working age population receiving incapacity benefit

**Table 3:** Summary of estimated marginal effects for pollution

Pollutant	Model type	1 μg/m^3^ increase			1 standard deviation increase		
		Covid-19 Cases	Hospital admissions	Deaths	Covid-19 Cases	Hospital admissions	Deaths
PM2.5	Negative binomial	9.4 (1.1, 17.7)	3.0 (0.43, 5.6)	2.3 (0.87, 3.6)	12.7 (1.5, 24.0)	4.1 (0.58, 7.6)	3.1 (1.2, 4.9)
	Negative binomial IV	10.3 (0.79, 19.8)	2.9 (0.36, 5.5)	2.2 (0.74, 3.7)	13.9 (1.1, 26.8)	3.9 (0.49, 7.4)	3.0 (1.0, 5.1)
	Spatial 50 km	15.1 (1.6, 28.5)	4.4 (0.55, 8.2)	2.8 (0.92, 4.6)	20.4 (2.2, 38.6)	5.9 (0.74, 11.1)	3.8 (1.2, 6.2)
	Spatial 100 km	14.9 (1.4, 28.4)	4.2 (0.54, 7.9)	2.8 (0.91, 4.6)	20.2 (1.9, 38.5)	5.7 (0.73, 10.7)	3.7 (1.2, 6.2)
	Spatial no cut-off	12.2 (1.9, 22.5)	3.3 (0.73, 5.9)	2.3 (0.80, 3.8)	16.5 (2.6, 30.5)	4.5 (0.99, 8.0)	3.1 (1.1, 5.2)
$\hbox {NO}_{{2}}$	Negative binomial	2.2 (0.2, 4.3)	–	0.35 (0.042, 0.66)	8.5 (0.77, 16.6)	–	1.4 (0.16, 2.5)

Note: Marginal effects are only reported if pollution is statistically significant at conventional levels (i.e. *p* < 0.05 or lower) in the initial estimation. Figures in parentheses are 95% confidence intervals. The table should be read as follows: A 1 μg/m^3^ increase in PM2.5 will increase Covid-19 cases by 10.3 according to the negative binomial IV model. Similarly, a 1 standard deviation increase in $\hbox {NO}_{{2}}$ will increase Covid-19 deaths by 1.4 according to the negative binomial model

Table 5 reports the results of the spatial econometric estimations based on Eq. 2 in which we include a spatially lagged dependent variable and spatial errors using three different spatial weight matrices (50 km, 100 km, no cut-off). Table 5 displays the estimated coefficients on $PM_{2.5}$ concentrations for the three dependent variables. The three models with differing weight matrices are presented. For each we also report the spatial error and spatial lag coefficients, $\lambda$ and $\rho$, respectively. Table 5 indicates that $PM_{2.5}$ concentrations continue to have a positive relationship with all three of the Covid-19 dependant variables, for all three of the spatial weight matrices.^26^ Where the estimated coefficients on $PM_{2.5}$ are not labelled as being statistically significant at 5% levels, they are significant at 10% levels. The introduction of the spatially lagged dependent variable into Eq. 2 changes the interpretation of our estimated coefficients. $PM_{2.5}$ concentrations in municipality *i* continue to affect the conditional mean of Covid-19 in that municipality but now that change in Covid-19 potentially changes the conditional mean of Covid-19 in other nearby municipalities (depending on our weight matrix). Furthermore, the change in Covid-19 in those nearby municipalities affects the conditional mean of Covid-19 in their neighbouring municipalities and so on. $PM_{2.5}$ concentrations therefore now have a *direct* effect on Covid-19 in their own municipality plus an *indirect* effect on Covid-19 in other municipalities.

**Table 4:** Instrumental variable results

VARIABLES	Covid-19 Cases	Hospital admissions	Deaths
	(1)	(2)	(3)
PM2.5, 5 year ave	0.12* (0.062)	0.14* (0.069)	0.23** (0.088)
First stage residuals (Durbin–Wu–Hausman)	− 0.027 (0.074)	0.012 (0.063)	0.0018 (0.10)
F-test on instruments (Prob> F)	190.48 (0.000)	190.48 (0.000)	190.48 (0.000)
Province Dummies	YES	YES	YES
Observations	355	355	355
Pseudo R^2^	0.17	0.19	0.18

Bootstrapped SE in parentheses, ***p < 0.001, **p < 0.01, *p < 0.05. Additional controls included in the estimations but not reported are Belgian border dummy, German border dummy, average household income, average household size, share of small housing, share of population under 18 years old, share of population over 70 years old, share of population who are smokers, population level, non-western migrant share, proximity to Schiphol airport, coastal dummy, average firm size, share of workers in elementary occupations, share of obesity, population density, share of working age population receiving incapacity benefit

If we take the example of our estimate of Covid-19 Cases using a weight matrix with a 50 km cut-off, which reports an estimated coefficient on $PM_{2.5}$ concentrations of 20.88, we can calculate the marginal effect to consist of a direct effect of 21.0, an indirect effect of -5.9, and a total effect of 15.1. This implies that a 1 unit change in $PM_{2.5}$ concentrations is associated with an increase in Covid-19 cases of 15.1. However, it is notable that the estimates of both $\lambda$ and $\rho$ from this model are not statistically significant, implying that spatial spillovers are not unduly influencing our results, either through the error term or through the dependent variable. Indeed, if we look at the estimates of $\lambda$ and $\rho$ from the other models in Table 5 we see that the majority are not statistically significant. This is particularly true of the spatial error coefficient $\lambda$. So, while we find limited evidence of statistically significant spatial spillovers, Table 5 at least confirms that our positive and statistically significant association between Covid-19 and $PM_{2.5}$ concentrations is robust to the inclusion of such spillovers.

**Table 5:** Sensitivity checks I—spatial results

	Covid cases	Hospital admissions	Deaths
*50 km cut off*			
PM2.5, 5 year ave	20.88* (10.69)	6.50* (3.25)	3.71* (1.49)
Spatial error ($\lambda )$	− 0.20 (0.52)	0.41 (0.35)	− 0.56 (0.59)
Spatial lag ($\rho )$	− 0.58 (0.29)	− 0.76* (0.31)	− 0.50 (0.29)
*100 km cut off*			
PM2.5, 5 year ave	21.40 (12.86)	6.57 (3.62)	4.00* (1.91)
Spatial error ($\lambda )$	− 0.54 (0.86)	0.42 (0.88)	− 0.90 (0.91)
Spatial lag ($\rho )$	− 0.57 (0.44)	− 0.74 (0.43)	− 0.57 (0.45)
*No distance cut off*			
PM2.5, 5 year ave	21.2 (12.55)	9.15* (4.19)	3.82* (1.87)
Spatial error ($\lambda )$	− 0.52 (1.05)	1.90** (0.41)	− 0.97 (1.17)
Spatial lag ($\rho )$	− 0.83 (0.62)	− 2.03** (0.64)	− 0.77 (0.64)
Observations	355	355	355

Robust SE in parentheses, ***p < 0.001, **p < 0.01, *p < 0.05. Additional controls included in the estimations but not reported are Belgian border dummy, German border dummy, average household income, average household size, share of small housing, share of population under 18 years old, share of population over 70 years old, share of population who are smokers, population level, non-western migrant share, proximity to Schiphol airport, coastal dummy, average firm size, share of workers in elementary occupations, share of obesity, population density, share of working age population receiving incapacity benefit

Finally, Tables 6 and 7 report some additional sensitivity estimations. In columns (1)–(3) of Table 6, rather than relying only on province dummies to control for the effect of the carnival, we include a municipality-level dummy of the seven most populous municipalities within North Brabant and Limburg provinces. In addition, we include a measure of the proximity of each municipality to the centroid of these seven combined municipalities. Table 6 indicates that these municipality-level carnival variables are not statistically significant, nor do they have the expected signs. Furthermore, the coefficients on $PM_{2.5}$ concentrations remain unaffected by their inclusion in terms of magnitude, sign, or significance.^27^

As mentioned earlier, the effect of urban density on the spread of Covid-19 has been an ongoing discussion with a number of studies providing mixed and inconclusive evidence. To explore this further, columns (4)–(6) in Table 6 report estimations in which we omitted from our analysis the major urban areas of Amsterdam, Rotterdam, Utrecht and The Hague in case, given their high levels of concentration, they are unduly influencing our results. The sign, significance and magnitude of our estimated coefficients on pollution are consistent with our previous results (in columns (1), (4) and (7) from Table 2).

**Table 6:** Sensitivity checks II—additional results

	Covid Cases (1)	Hospital admissions (2)	Deaths (3)	Covid Cases (4)	Hospital admissions (5)	Deaths (6)
PM2.5, 5 year ave	0.12* (0.051)	0.15* (0.066)	0.25** (0.071)	0.13** (0.048)	0.17** (0.063)	0.27*** (0.068)
Carnival(municip)	− 0.23 (0.14)	− 0.12 (0.17)	− 0.39 (0.22)			
Proximity to Carnival(municip)	− 1126 (754)	− 374 (1039)	− 1598 (1238)			
Belgian border	0.13 (0.11)	− 0.100 (0.11)	0.016 (0.15)	0.1 (0.11)	− 0.068 (0.11)	0.094 (0.15)
German border	− 0.30*** (0.084)	− 0.25** (0.080)	− 0.37*** (0.11)	− 0.30*** (0.079)	− 0.26*** (0.081)	− 0.37*** (0.11)
Ave gross income	− 0.029* (0.012)	− 0.029* (0.013)	− 0.051* (0.023)	− 0.024* (0.011)	− 0.031* (0.013)	− 0.045 (0.024)
Ave household size	1.78*** (0.38)	2.20*** (0.50)	1.98** (0.69)	1.39*** (0.42)	1.67** (0.53)	1.28 (0.73)
Sh of small houses	1.25*** (0.26)	0.68* (0.28)	0.91* (0.37)	1.23*** (0.25)	0.51 (0.27)	0.85* (0.34)
Sh of under 18	− 2.85 (2.30)	− 7.81** (2.89)	− 1.90 (3.78)	− 1.33 (2.57)	− 5.03 (3.18)	− 0.36 (4.10)
Sh of 70+	4.86*** (1.50)	2.10 (1.65)	9.64*** (2.32)	4.66** (1.53)	2.25 (1.58)	8.23*** (2.42)
Smokers	0.060*** (0.017)	0.034* (0.016)	0.067** (0.022)	0.052** (0.019)	0.023 (0.017)	0.050* (0.025)
ln (population)	1.01*** (0.044)	1.01*** (0.058)	1.07*** (0.075)	0.98*** (0.043)	0.98*** (0.056)	1.00*** (0.070)
Non-western mig sh	1.13* (0.67)	1.70** (0.64)	1.75 (1.02)	0.022 (0.89)	0.53 (0.83)	− 0.40 (1.28)
Observations	355	355	355	351	351	351
Province Dummies	YES	YES	YES	YES	YES	YES
Pseudo R^2^	0.17	0.19	0.19	0.16	0.18	0.18

Robust SE in parentheses ***p < 0.001, **p < 0.01, *p < 0.05. Additional controls not reported are proximity to Schiphol airport, coastal dummy, average firm size, share of population receiving incapacity benefits, share of workers in elementary occupations, share of obesity, population density. Columns (1)–(3) include additional carnival variables while columns (4)–(6) omit the four major cities of Amsterdam, Rotterdam, Utrecht and The Hague

Finally, in columns (1)–(3) of Table 7 we replace our 5 year average of $PM_{2.5}$ with a 10 year average. As can be seen, all three coefficients on $PM_{2.5}$ remain positive, statistically significant and of almost identical magnitude to those in Table 2. This suggests that our results are not at all sensitive to precisely how we measure long term pollution exposure. In columns (4)–(6) of Table 7 we return to 5 year pollution averages but include all three pollutants together in each estimation. The 3 pollutants are highly correlated with the highest correlation of 0.79 between $PM_{2.5}$ and $NO_{2}$. This raises question marks over the precision with which the coefficients on these pollutants are estimated but, nevertheless, we see that when all 3 pollutants are included together, $PM_{2.5}$ generally remains statistically significant while the other pollutants are not significant.^28^

**Table 7:** Sensitivity checks III—additional results

	Covid cases (1)	Hospital admissions (2)	Deaths (3)	Covid cases (4)	Hospital admissions (5)	Deaths (6)
PM2.5, 10 year ave	0.12** (0.044)	0.13* (0.055)	0.22*** (0.064)			
PM2.5, 5 year ave				0.093 (0.064)	0.19* (0.082)	0.26** (0.095)
NO2, 5 year ave				0.0062 (0.017)	− 0.022 (0.020)	− 0.024 (0.024)
SO2, 5 year ave				0.052 (0.086)	0.043 (0.081)	0.15 (0.12)
Belgian border	0.17 (0.11)	− 0.092 (0.11)	0.068 (0.16)	0.18 (0.11)	− 0.081 (0.11)	0.076 (0.15)
German border	− 0.30*** (0.084)	− 0.25** (0.080)	− 0.36*** (0.11)	− 0.30*** (0.083)	− 0.25** (0.083)	− 0.37*** (0.11)
Ave gross income	− 0.029* (0.012)	− 0.028* (0.013)	− 0.049* (0.022)	− 0.026* (0.011)	− 0.028* (0.013)	− 0.046* (0.022)
Ave household size	1.79*** (0.37)	2.17*** (0.49)	2.00** (0.69)	1.79*** (0.38)	2.19*** (0.49)	2.00** (0.67)
Sh of small houses	1.22*** (0.27)	0.65* (0.28)	0.84* (0.40)	1.27*** (0.26)	0.67* (0.28)	0.91** (0.35)
Sh of pop under 18	− 3.01 (2.26)	− 7.70** (2.79)	− 2.42 (3.76)	− 3.17 (2.28)	− 8.05** (2.82)	− 2.61 (3.70)
Sh of pop 70+	4.91** (1.51)	2.13 (1.61)	9.46*** (2.26)	4.47** (1.51)	1.78 (1.65)	8.85*** (2.32)
Smokers	0.058*** (0.016)	0.032* (0.015)	0.063** (0.021)	0.056*** (0.017)	0.034* (0.016)	0.062** (0.021)
ln (population)	1.00*** (0.040)	1.00*** (0.052)	1.02*** (0.065)	1.00*** (0.042)	1.00*** (0.053)	1.03*** (0.069)
Non-western mig sh	1.42* (0.67)	1.91** (0.62)	2.35* (1.07)	1.25 (0.66)	1.90** (0.61)	2.07* (0.98)
Observations	355	355	355	355	355	355
Province Dummies	YES	YES	YES	YES	YES	YES
Pseudo R^2^	0.17	0.19	0.18	0.17	0.19	0.19

Robust SE in parentheses ***p < 0.001, **p < 0.01, *p < 0.05. Additional controls not reported are proximity to Schiphol airport, coastal dummy, average firm size, share of population receiving incapacity benefits, share of workers in elementary occupations, share of obesity, population density. Columns (1)–(3) include a 10 year average of PM2.5 rather than 5 year, while columns (4)–(6) include all three pollutants together

## Conclusion

This paper has contributed to the nascent literature examining the link between poor air quality and Covid-19. We examine data for 355 Dutch municipalities to identify the relationship between concentrations of $PM_{2.5}$, $NO_{2}$ and $SO_{2}$ and Covid-19 cases, hospital admissions and deaths. In contrast to much of the previous literature we are able to control for a wide range of potential confounding effects and, by examining Covid-19 data between February and June 2020, are able to examine almost the full wave of the epidemic within the Netherlands.

We find compelling evidence of a statistically significant positive relationship between air pollution and Covid-19 cases, hospital admissions and deaths. This relationship is particularly evident for concentrations of $PM_{2.5}$ and to a lesser extent $NO_{2}$ and persists after controlling for explanatory variables capturing income, demography, social and physical proximity, employment/education, health and spatial factors. The relationship withstands a number of sensitivity and robustness exercises including instrumenting pollution to mitigate some forms of potential endogeneity and modelling spatial spillovers using spatial econometric techniques.

Our results indicate that, on average, and other things being equal, a municipality with 1 μg/m^3^ more $PM_{2.5}$ concentrations than another will have between 9.4 and 15.1 more Covid-19 cases, depending on our model. It will also have between 2.9 and 4.4 more Covid-19 hospital admissions and between 2.2 and 2.8 more Covid-19 deaths. The only comparable study to our own is provided by Wu et al. (2020) who examine Covid-19 deaths in the US. They find that a 1 μg/m^3^ increase in $PM_{2.5}$ is associated with an 8% increase in the Covid-19 death rate. Since the mean number of deaths in our sample is 16.86, our estimated increases of between 2.2 and 2.8 are equivalent to increases of between 13.0% and 16.6%, which are clearly larger in magnitude than those of Wu et al. (2020).

It is notable that while we find $PM_{2.5}$ and, to a lesser extent, $NO_{2}$ to be associated with Covid-19 outcomes, the same is not true of $SO_{2}$ concentrations. The most likely explanation for this difference is that, unlike $PM_{2.5}$ and $NO_{2}$, $SO_{2}$ concentrations have fallen dramatically in recent years. For instance, $SO_{2}$ concentrations from regional background monitoring stations have decreased from 5 to 15 μg/m^3^ to an average of 1 μg/m^3^ since 1990.^29^ Concentrations of $PM_{2.5}$ and $NO_{2}$ have not experienced similar declines. Furthermore, the EU limit value for $SO_{2}$ has not been exceeded anywhere in the Netherlands since 1998. Again, the same is not true of $PM_{2.5}$ and $NO_{2}$ concentrations. If $SO_{2}$ concentrations are not sufficiently high to be causing adverse health impacts then they would appear to be less likely to contribute to Covid-19 outcomes.

While our findings remain correlative rather than causal, there are plausible mechanisms through which air pollution could be affecting Covid-19 outcomes. Given the existing evidence of a link between exposure to air pollution and a persistent inflammatory response within the respiratory tract (Abbey et al. 1999; De Weerdt et al. 2020; Conticini et al. 2020), it is possible that individuals who have experienced long term pollution exposure will face a higher risk of hospitalisation and death upon contracting Covid-19. Furthermore, since it has also been claimed that exposure to pollution can increase the risk of infection by viruses that target the respiratory tract (Travaglio et al. 2020; Conticini et al. 2020), and that particulate matter might actually carry the Covid-19 virus (Setti et al. 2020), it is also feasible that pollution exposure could increase the number of actual Covid-19 cases.

Two clear policy implications arise from our analysis. First, the impact of poor air quality on Covid-19 morbidity and mortality represents a considerable and unexpected additional cost from air pollution. Our results would therefore suggest that more stringent air pollution regulation may be required, even in a relatively well-regulated nation such as the Netherlands. Second, our findings should prove useful to public health officials by signaling where subsequent waves of Covid-19, or indeed future pandemics of respiratory disease, might hit hardest. This could be particularly important in countries where pollution sources don’t correlate well with major metropolitan areas. If, for instance, livestock production is a major source of emissions, as may be the case in some parts of the Netherlands, then advance warning may prove particularly beneficial for rural areas where healthcare infrastructure and coordination may be less developed.

We believe the statistical relationships we have observed between $PM_{2.5}$ concentrations and Covid-19 data are robust. Furthermore, given the established literature linking poor air quality with respiratory disease, the existence of a similar link between poor air quality and Covid-19 outcomes is plausible. By focusing on the Netherlands we can also be confident that the correlations we find between $PM_{2.5}$ and Covid-19 outcomes are not simply a result of Covid-19 cases being clustered in large cities where pollution may be higher, as may be the case in Northern Italy for instance. As we have noted, Covid-19 hotspots in the Netherlands were in relatively rural regions rather than the big cities. Nevertheless, a degree of caution is needed. We do not claim to have found a causal relationship between pollution and Covid-19 outcomes. Instead, we have found correlations that persist even when a wide range of control variables are included and a number of different estimation methods are utilised. Until detailed individual-level data is available providing information on Covid-19 and a wide range of other individual characteristics the statistical evidence of a link between air quality and Covid-19 outcomes will remain suggestive rather than conclusive.

## Figures and Tables

**Fig. 2:**
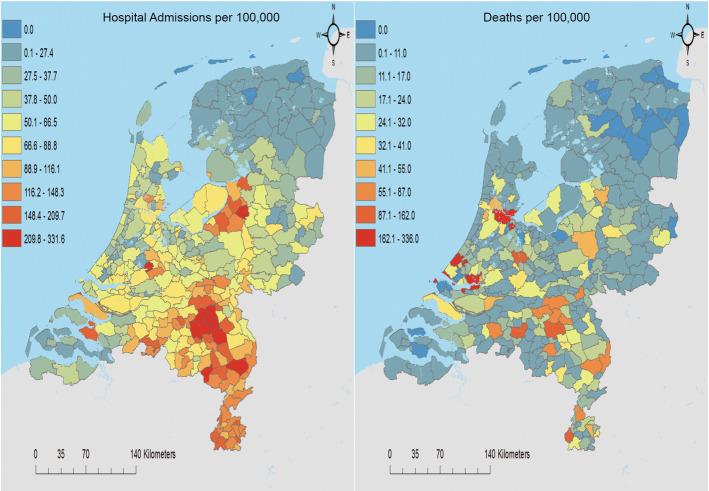
Covid-19 hospital admissions and deaths per 100,000 people

## Appendix

See Figs. 2 and 3, Tables 8, 9, 9, 10, 11 and 12.

**Table 8:** First stage results for PM2.5

	PM2.5 5 year ave
Ave commuting time	0.046*** (0.012)
Ave commuting time squared	− 0.00031*** (0.000096)
PM 5 year ave (1995–2000)	0.35*** (0.015)
Proximity to Schiphol	0.0022 (0.0012)
Belgian border	− 0.37** (0.14)
Coast	− 0.92*** (0.13)
German border	− 0.068 (0.13)
Ave gross income	0.033* (0.016)
ln (firm size)	− 0.12 (0.076)
Sh elementary occup	− 1.59 (1.10)
Sh high educated	1.29* (0.52)
Ave household size	0.77 (0.57)
Sh of small houses	0.30 (0.78)
Sh of pop under 18	− 8.40* (3.49)
Sh of pop 70+	− 8.29*** (2.22)
Obesity	0.012 (0.023)
Smokers	− 0.055* (0.025)
Sh on incapacity benefits	9.31*** (2.82)
Pop density	0.00010* (0.000051)
ln (population)	0.049 (0.059)
Sh non-western migrants	− 3.76*** (1.16)
Observations	355 0.821
R^2^	

SE in parentheses. ****p* < 0.001, ***p* < 0.01, **p* < 0.05

**Table 9:** Instrumental variable results for $\hbox {NO}_{\mathrm {2}}$

VARIABLES	Covid-19 Cases	Hospital admissions	Deaths
	(1)	(2)	(3)
NO2, 5 year ave	0.035* (0.018)	0.013 (0.019)	0.027 (0.026)
First stage residuals (Durbin–Wu–Hausman)	− 0.026 (0.032)	0.0057 (0.034)	0.026 (0.051)
F-test on instruments (Prob> F)	353.89 (0.000)	353.89 (0.000)	353.89 (0.000)
Province dummies	YES	YES	YES
Observations	355	355	355
Pseudo R^2^	0.17	0.19	0.18

Bootstrapped SE in parentheses, ****p* < 0.001; ***p* < 0.01; **p* < 0.05. Additional controls included in the estimations but not reported are Belgian border dummy, German border dummy, average household income, average household size, share of small housing, share of population under 18 years old, share of population over 70 years old, share of population who are smokers, population level, non-western migrant share, proximity to Schiphol airport, coastal dummy, average firm size, share of workers in elementary occupations, share of obesity, population density, share of working age population receiving incapacity benefit

**Table 10:** Instrumental variable results for $\hbox {SO}_{\mathrm {2}}$

VARIABLES	Covid-19 Cases	Hospital admissions	Deaths
	(1)	(2)	(3)
SO2, 5 year ave	0.42* (0.20)	0.20 (0.22)	0.30 (0.32)
First stage residuals (Durbin–Wu–Hausman)	− 0.39 (0.22)	− 0.18 (0.25)	− 0.14 (0.36)
F-test on instruments (Prob> F)	71.17	71.17	71.17
	(0.000)	(0.000)	(0.000)
Province dummies	YES	YES	YES
Observations	355	355	355
Pseudo R^2^	0.17	0.19	0.18

Bootstrapped SE in parentheses, ****p* < 0.001; ***p* < 0.01, **p* < 0.05. Additional controls included in the estimations but not reported are Belgian border dummy, German border dummy, average household income, average household size, share of small housing, share of population under 18 years old, share of population over 70 years old, share of population who are smokers, population level, non-western migrant share, proximity to Schiphol airport, coastal dummy, average firm size, share of workers in elementary occupations, share of obesity, population density, share of working age population receiving incapacity benefit

**Table 11:** Spatial econometric results for $\hbox {NO}_{\mathrm {2}}$

	Covid cases	Hospital admissions	Deaths
*50 km cut off*			
NO2, 5 year ave	3.75 (5.26)	1.00 (1.37)	0.37 (0.82)
Spatial error ($\lambda )$	− 0.060 (0.55)	0.57 (0.34)	− 0.56 (0.83)
Spatial lag ($\rho )$	− 0.53 (0.35)	− 0.66 (0.37)	− 0.28 (0.44)
*100 km cut off*			
NO2, 5 year ave	− 6.1 (4.9)	− 0.49 (2.12)	− 0.97 (0.61)
Spatial error ($\lambda )$	− 2.20 (1.21)	0.16 (1.25)	− 2.92*** (1.02)
Spatial lag ($\rho )$	0.41 (0.39)	− 0.11 (0.72)	0.61* (0.29)
*No distance cut off*			
NO2, 5 year ave	− 3.80 (5.34)	− 0.087 (1.64)	− 1.71** (0.53)
Spatial error ($\lambda )$	− 2.21 (1.65)	0.20 (0.99)	− 5.12*** (0.99)
Spatial lag ($\rho )$	0.30 (0.64)	− 0.41 (0.75)	1.67*** (0.32)
Observations	355	355	355

Robust SE in parentheses, ****p* < 0.001; ***p* < 0.01; **p* < 0.05. Additional controls included in the estimations but not reported are Belgian border dummy, German border dummy, average household income, average household size, share of small housing, share of population under 18 years old, share of population over 70 years old, share of population who are smokers, population level, non-western migrant share, proximity to Schiphol airport, coastal dummy, average firm size, share of workers in elementary occupations, share of obesity, population density, share of working age population receiving incapacity benefit

**Table 12:** Spatial econometric results for $\hbox {SO}_{\mathrm {2}}$

	Covid cases	Hospital admissions	Deaths
*50 km cut off*			
SO2, 5 year ave	− 12.91 (33.26)	− 10.96 (8.84)	0.035 (4.53)
Spatial error ($\lambda )$	− 0.24 (0.54)	0.37 (0.39)	− 0.78 (0.71)
Spatial lag ($\rho )$	− 0.31 (0.28)	− 0.30 (0.31)	− 0.13 (0.29)
*100 km cut off*			
SO2, 5 year ave	− 33.57 (29.76)	− 15.32 (8.1)	− 2.74 (4.11)
Spatial error ($\lambda )$	− 1.17 (0.94)	0.00096 (0.00)	− 1.93* (0.97)
Spatial lag ($\rho )$	0.13 (0.32)	0.091 (0.34)	0.28 (0.29)
*No distance cut off*			
SO2, 5 year ave	− 25.59 (30.95)	− 13.84 (8.2)	− 1.51 (4.2)
Spatial error ($\lambda )$	− 1.37 (1.20)	− 0.063 (0.84)	− 2.41* (1.2)
Spatial lag ($\rho )$	0.054 (0.48)	− 0.018 (0.48)	0.31 (0.41)
Observations	355	355	355

Robust SE in parentheses, ****p* < 0.001, ***p* < 0.01, **p* < 0.05. Additional controls included in the estimations but not reported are Belgian border dummy, German border dummy, average household income, average household size, share of small housing, share of population under 18 years old, share of population over 70 years old, share of population who are smokers, population level, non-western migrant share, proximity to Schiphol airport, coastal dummy, average firm size, share of workers in elementary occupations, share of obesity, population density, share of working age population receiving incapacity benefit

## Footnotes

1 As of June 9th 2020. Source https://www.worldometers.info/coronavirus/#countries.

2 It also ideally requires an experimental setting in which some individuals are exposed to pollution while others are not.

3 The average US county-level population is 104,435 and average area of each county is 3130 km^2^ (US Census Bureau). In contrast, the average population in Dutch municipalities is 48,682 while the average area is 94.8 km^2^.

4 As of June 9th 2020. All Dutch Covid-19 data is provided by the National Institute for Public Health and the Environment (RIVM). The international ranking excludes the principalities of San Marino and Andorra and stems from: https://www.worldometers.info/coronavirus/#countries.

5 Equivalent to 137.5 cases per million of population in North Brabant and 95.3 per million in South Holland.

6 Equivalent to 68.5 cases per million of population in Groningen and 81.3 cases per million in Drenthe.

7 Figure 2 in the “Appendix” shows that both hospital admissions and deaths per capita from Covid-19 have also been disproportionately high in these south eastern regions.

8 A non-exhaustive list includes: https://www.nrc.nl/nieuws/2020/04/09/vuile-lucht-vergroot-de-kans-om-te-sterven-aan-covid-19-a3996388https://nos.nl/nieuwsuur/artikel/2332460-gebieden-met-veel-luchtverontreiniging-zwaarder-getroffen-door-corona.htmlhttps://www.rtlnieuws.nl/nieuws/nederland/artikel/5094341/corona-update-noorden-minder-besmettingenhttps://nadavos.nl/nieuws/2020-luchtverontreiniging-covid-19https://www.trouw.nl/binnenland/zorgt-de-veehouderij-voor-meer-coronadoden-in-oost-brabant~b2b5487d/.

9 2019 data from CBS Statline.

10 This article in Trouw, a Dutch national newspaper, expresses the concerns of local residents that poor air quality in the intensively livestocked regions may be leaving the local population susceptible to Covid-19, https://www.trouw.nl/binnenland/zorgt-de-veehouderij-voor-meer-coronadoden-in-oost-brabant~b2b5487d/

11 We bootstrap the standard errors in the second stage using 500 replications.

12 Access to these data sets requires a confidentiality agreement with Statistics Netherlands.

13 See Koster and Ozgen (2020) for more details. Generally, for short distances (e.g. less than 5 km) it is slower to use the road network and so the Euclidean travel time is used.

14 Our analysis does not control for meteorological variables such as temperature and humidity. The evidence that such variables can affect Covid-19 is very limited, for instance Wu et al. (2020) found no statistically significant impact of meteorological variables on US Covid-19 death rates. In addition, meteorological data does not appear to be available at anything close to municipal-level resolution. For example, Copernicus data is only available at 0.25 degree resolution, which would provide approximately 23 observations for the Netherlands. Perhaps more fundamentally, there is very little variation in meteorological conditions within the Netherlands due to its relatively small size and lack of variation in altitude. We therefore rely on our province fixed effects to capture any variation in meteorological conditions.

15 https://www.ggd.amsterdam.nl/coronavirus/.

16 https://www.rivm.nl/coronavirus-covid-19/actueel.

17 The fact that Covid-19 deaths in the Netherlands peaked several days prior to the number of Covid-19 cases, as discussed in Sect. 2, may be evidence of reporting difficulties. Alternatively, it may simply reflect the growing number of tests being performed and an increasing share of minor cases being detected.

18 For more details on the construction of the pollution concentrations data see Velders et al. (2017).

19 In unreported estimations we include $NH_{3}$ as an explanatory variable. If included instead of $PM_{2.5}$ it is generally statistically significant. When included alongside $PM_{2.5}$ it becomes insignificant while $PM_{2.5}$ is significant.

20 See Kirby (2020) and Platt and Warwick (2020) https://www.ifs.org.uk/inequality/chapter/are-some-ethnic-groups-more-vulnerable-to-covid-19-than-others.

21 https://www.theguardian.com/world/2020/may/11/manual-workers-likelier-to-die-from-covid-19-than-professionals.

22 See for example, https://www.centreforcities.org/blog/have-uk-cities-been-hotbeds-of-covid-19-pandemic/http://jedkolko.com/2020/04/15/where-covid19-death-rates-are-highest/.

23 We don’t have the average size of housing as Statistics Netherlands provides house sizes in categories.

24 An average value of 33 hospital admissions per municipality is equivalent to 677.8 per million of population, while the average value of 17 deaths per municipality is equivalent to 349.2 per million.

25 The corresponding $\chi ^2$ statistics were 27.3, 17.0, and 10.7, respectively.

26 Fisher combination tests confirmed that $PM_{2.5}$ continued to have an impact on Covid-19 outcomes, regardless of the spatial cut-off chosen.

27 A Fisher combination test across the three specifications produced a $\chi ^2$ statistic of 21.9.

28 Unreported sensitivity analyses test alternative measures of many of our explanatory variables. For instance, we replaced population density with the share of urban land, average household income with household income of the poorest 10%, the share of workers in elementary occupations with the share of workers in routine task-intensive occupations and others. Results were generally similar and, importantly, the sign, significance and magnitude of the pollution coefficients were not at all sensitive to the choice of explanatory variables.

29 https://www.clo.nl/indicatoren/nl0441-zwaveldioxide.
